# Immune Checkpoint Molecules and Glucose Metabolism in HIV-Induced T Cell Exhaustion

**DOI:** 10.3390/biomedicines10112809

**Published:** 2022-11-04

**Authors:** Yee Teng Chan, Heng Choon Cheong, Ting Fang Tang, Reena Rajasuriar, Kian-Kai Cheng, Chung Yeng Looi, Won Fen Wong, Adeeba Kamarulzaman

**Affiliations:** 1 Department of Medical Microbiology, Faculty of Medicine, University of Malaya, Kuala Lumpur 50603, Malaysia; yeeteng111@gmail.com (Y.T.C.); cheonghengchoon@gmail.com (H.C.C.); tiffanytftang@gmail.com (T.F.T.); 2 Department of Medicine, Faculty of Medicine, University of Malaya, Kuala Lumpur 50603, Malaysia; reena@um.edu.my (R.R.); adeeba@um.edu.my (A.K.); 3 Centre of Excellence for Research in AIDS (CERiA), University of Malaya, Kuala Lumpur 50603, Malaysia; 4 Innovation Centre in Agritechnology (ICA), Universiti Teknologi Malaysia, Pagoh 84600, Malaysia; chengkiankai@utm.my; 5 School of Bioscience, Taylor’s University, Subang Jaya 47500, Malaysia; chungyeng.looi@taylors.edu.my

**Keywords:** CTLA-4, HIV, glucose, metabolism, PD-1, T cell exhaustion

## Abstract

The progressive decline of CD8^+^ cytotoxic T cells in human immunodeficiency virus (HIV)-infected patients due to infection-triggered cell exhaustion and cell death is significantly correlated with disease severity and progression into the life-threatening acquired immunodeficiency syndrome (AIDS) stage. T cell exhaustion is a condition of cell dysfunction despite antigen engagement, characterized by augmented surface expression of immune checkpoint molecules such as programmed cell death protein 1 (PD-1), which suppress T cell receptor (TCR) signaling and negatively impact the proliferative and effector activities of T cells. T cell function is tightly modulated by cellular glucose metabolism, which produces adequate energy to support a robust reaction when battling pathogen infection. The transition of the T cells from an active to an exhausted state following pathogen persistence involves a drastic change in metabolic activity. This review highlights the interplay between immune checkpoint molecules and glucose metabolism that contributes to T cell exhaustion in the context of chronic HIV infection, which could deliver an insight into the rational design of a novel therapeutic strategy.

## 1. Introduction

The human immunodeficiency virus (HIV) is a human pathogen that attacks the immune system by targeting CD4^+^ T lymphocytes. HIV infection can result in acquired immunodeficiency syndrome (AIDS), a fatal stage at which the host immune system collapses and becomes vulnerable to many types of opportunistic infections. There is currently no effective cure or prophylactic vaccine for HIV. However, highly active antiretroviral therapy (HAART), which comprises a combination of at least three antiretroviral regimens of different classes such as nucleoside reverse transcriptase inhibitors (typically tenofovir), non-nucleoside reverse transcriptase inhibitors, or protease inhibitors, has significantly increased the lifespan of people living with HIV (PLWH). Nonetheless, low level of viral replication persists even in aviremic PLWH due to latently infected cells; hence, a lifelong treatment is necessary to prevent HIV rebound. Early HIV infection elicits robust T cell-mediated responses, but such antiviral responses wane as the infection persists. This is caused by a spectrum of functional defects in CD8^+^ T cells or exhaustion that arises following chronic antigen exposure [1,2].

T cells are the centerpiece in the field of immune checkpoint-based immunotherapy, which is widely applied in clinics nowadays. Immune checkpoint blockade inhibits the negative signal in T cells and thus results in active effector functions, i.e., secretion of cytokines and cytotoxic elimination of target cells. Immune checkpoint-based immunotherapy has been shown to be an effective therapeutic treatment for tumor malignancies, though less so in clinical trials involving infectious diseases such as HIV, hepatitis B virus (HBV), and hepatitis C virus (HCV) infections, particularly for reviving T cell activity. Studies have reported varying degrees of suboptimal T cell activity and poor durability in a simian immunodeficiency virus (SIV)-infected rhesus macaque model [3], as well as in case reports among PLWH [4]. Most PLWH could only regenerate a limited number of functional T cells below the immuno-threshold, hence the clinical use of immune checkpoint-based immunotherapy remains questionable in the infection setting [5]. This suggests that the immune checkpoint blockade alone might not be adequate to reprogram the chromatin landscape of exhausted T cells, and this necessitates the development of other potent interventions that could complement the treatment’s efficacy and efficiency in HIV eradication [6,7,8]. 

Immunometabolism is an emerging field that decrypts the mechanism of the cellular metabolic program to produce energy for robust activities when immune cells are triggered by internal or external stimuli such as pathogen infection [9]. In recent years, a substantial number of studies have attempted to uncover the complexity of the mechanism that underlies the immunometabolism process, and a new therapeutic perspective through manipulating immunometabolism has been proposed in the hope of reversing T cell exhaustion and boosting the host immune response to HIV. During chronic HIV infection, T cell exhaustion is accompanied by both elevated expression of immune checkpoint molecules and altered intrinsic metabolic programming, implying a close connection between these two events. A tilt in the balance between the two could disrupt T cell performance and provoke immune dysregulation. Hence, additional therapeutic options that take into consideration both immune checkpoint inhibitors and immunometabolism boosting drugs may be ideal to complement the current HIV treatment. This review discusses the interplay of checkpoint molecules and glucose metabolism that is associated with T cell exhaustion in HIV infection. 

## 2. Elevated Expression of T Cell Immune Checkpoint Molecules in HIV 

The concept of T cell exhaustion was first introduced in the 1990s by Moskophidis and colleagues as they observed the disappearance and dysfunction of the CD8^+^ T lymphocyte in a mouse model following infection with noncytopathic strains of chronic lymphocytic choriomeningitis virus (LCMV) [10]. Following this discovery, various studies have focused on elucidating the implication of T cell exhaustion in human diseases, particularly tumor progression and chronic infections by various pathogens such as HIV, HBV, and HCV. Cellular exhaustion of CD8^+^ T cells is characterized by impaired capability in cell proliferation, cytokine production, and effector activity, accompanied by increased expression of immune checkpoint molecules [11,12,13,14,15,16]. At present, the term “cellular exhaustion” is no longer restricted to CD8^+^ T cells, as similar observations have been reported in CD4^+^ T lymphocytes and other immune cells [17,18]. 

To tackle the immune dysfunction following T cell exhaustion in viral infection, many research attempts focus on reversing exhaustion and reinvigorating T cell function by administration of an immune checkpoint blockade. Immune checkpoint molecules that are directly associated with T cell exhaustion and have shown increased expression levels during chronic HIV infection include programmed cell death protein 1 (PD-1), cytotoxic T-lymphocyte antigen-4 (CTLA-4), T cell immunoglobulin and mucin domain-containing protein 3 (TIM-3), lymphocyte-activation gene 3 (LAG-3), and T cell immunoreceptor with immunoglobulin and ITIM domains (TIGIT). An association between these checkpoint inhibitors and HIV infection is discussed below. 

### 2.1. PD-1

PD-1 receptor is the most extensively studied exhaustion factor that exerts a negative impact on T cells through binding to programmed death-ligand 1 (PD-L1) or PD-L2 [19]. The cytoplasmic domain of PD-1 possesses an immunotyrosine inhibitory motif (ITIM) and an immunotyrosine switch motif (ITSM) that recruit and dephosphorylate Src homology 2-domain-containing tyrosine phosphatase 2 (SHP-2), a key signal transducer of T cell receptor (TCR) signaling [20,21]. These negative regulatory motifs are important to the cell physiology as they transduce inhibitory signals that inactivate the effector T cells, which counteracts the TCR signaling. Although this negative signal is important to prevent cell hyperactivation, an elevated expression of PD-1 in effector cells during chronic viral infections results in an undesired outcome that dampens effector activity whilst viral antigens persist in the host [11,22]. 

Viruses exploit PD-1 mediated suppression to evade immune surveillance and sustain their replication in the host cells. In chronic LCMV infection, increased PD-1 mediates CD8^+^ T cell exhaustion, whereas therapy targeting PD-1 restores T cell function and proliferation [22,23,24]. In the case of HIV infection, the HIV Tat protein induces the expression of PD-L1 via tumor necrosis factor-alpha (TNF-α) and toll-like receptor 4 (TLR4), and this negatively affects the ability of dendritic cells to recruit T cells [25]. The HIV Nef protein drives the upregulation of PD-1 during in vitro infection through its proline-rich motif and the activation of p38 signaling pathway [26]. PD-1 overexpression on CD8^+^ T cells is correlated with HIV viral load and disease progression [27]. During the viremia stage, HIV-specific CD8^+^ T cells exhibit PD-1 upregulation and exhaustion phenotypes, which could be reverted by PD-1 blockade [11,28]. 

Inhibition of the PD-1 and PD-L1 axis using in vitro and in vivo experiments contributes to the recovery of T cell functions [29,30,31] and potentially reverses latency in PLWH receiving HAART [32]. Nonetheless, PD-1 and PD-L1 inhibitors are currently approved only for cancer immunotherapy owing to encouraging treatment results obtained from clinical trials, but not for chronic HIV infections [33,34,35]. To date, clinical trials have suggested treatment with PD-1 inhibitors enhances the HIV-specific CD8^+^ response, but none has shown promise in wiping out the viral reservoir, and there is concern over immune-mediated toxicities [36,37]. A recent study demonstrates no reduction in HIV reservoir among PLWH who received anti-PD-1 drugs [38]. This suggests that the blockade of the PD-1/PD-L1 axis alone might be insufficient to tackle the HIV reservoir. Perhaps a combination therapy strategy that includes other components such as cell metabolism enhancement should be assessed to achieve viral eradication without compromising the safety of PLWH. 

### 2.2. CTLA-4

CTLA-4 is another important negative regulator of T cell function that is upregulated during chronic viral infections and tumor growth. Upon its interaction with CD80 and CD86 ligands, CTLA-4 transduces a signal through the serine-threonine protein phosphatase 2A to inhibit nuclear factor of activated T cell (NFAT) nuclear translocation, thereby inhibiting interleukin-2 (IL-2) production and cell proliferation [39]. CTLA-4 hinders the virus-specific CD4^+^ and CD8^+^ T cell activities in chronic HIV infections despite HAART prescription [14,40], resulting in weak immune responses and a poor disease prognosis. 

CTLA-4 is upregulated in HIV-specific CD4^+^ T cells in untreated PLWH of viremic controllers with acute and chronic infections, excluding the elite controllers [14]. The level of CTLA-4 is negatively correlated with IL-2 production but positively correlated with HIV disease progression. HIV preferentially infects CTLA^+^ CD4^+^ T cells in vitro and negatively modulates CTLA-4 expression in the presence of Nef protein to allow productive viral replication [41]. A study using HAART-treated SIV-infected rhesus macaques uncovers that memory CD4^+^ T cells expressing CTLA-4 contain high levels of SIV DNA and infectious virus, suggesting CTLA-4 could be an additional target for eliminating the viral reservoir [42].

FoxP3^+^ CD8^+^ regulatory T (Treg) cells expressing a high level of CTLA-4 are found to have no cytolytic potential and are directly correlated with high viremia in SIV-infected rhesus macaques [43]. A similar observation of increased CTLA-4^+^ FoxP3^+^ CD8^+^ Treg cells in untreated PLWH and early initiation of HAART reduces this immunosuppressive population [44]. The same study also reports the elite controllers presenting a similar level of CTLA-4 on FoxP3^+^ CD8^+^ T cells as the HIV-negative individuals, which could be associated with the maintenance of T cell antiviral responses [44].

CTLA-4 blockade in vitro leads to an increase in HIV-specific CD4^+^ T cell function [14]. A SIV infection in a macaque model demonstrates an increase in T cell activation and latency reversal upon CTLA-4 blockade [45]. Anti-CTLA-4 treatment in PLWH on HAART results in increased CD4^+^ T cell activation and a decline in plasma HIV RNA [46]. Additionally, CD8^+^ T cells with HLA-B*35Px restriction display CTLA-4 upregulation and acquire a functionally impaired phenotype that is reversible by in vitro anti-CTLA-4 treatment [47].

Dual blockade of PD-1 and CTLA-4 in vitro demonstrates synergistic effects and latent reversal in the proliferating CD4^+^ T cells [48]. A recent clinical trial has shown a positive outcome of anti-PD-1 and anti-CTLA-4 antibodies in the clearance of the HIV reservoir in PLWH with advanced malignancies, highlighting their potential applications in HIV treatment [38]. 

### 2.3. TIM-3

TIM-3 was initially identified as a T helper 1 (Th1)-specific transmembrane protein that characterizes the differentiated Th1 CD4^+^ T cell [49]. TIM-3 regulates T cell proliferation, production of pro-inflammatory cytokines, interferon-gamma (IFN-γ), and peripheral tolerance [50], which are driven by the binding of galectin-9, a ligand of TIM-3 [51]. The TIM-3/galectin-9 pathway triggers inhibitory signaling, reduces IFN-γ producing Th1 cells, and induces cell death in activated Th1 cells [51].

During HIV infection, viral proteins such as Nef and Vpu exert opposing effects on the TIM-3 expression level in infected CD4^+^ T cells. As shown by a recent in vitro study, the HIV-1 Nef protein mediates the upregulation of TIM-3 through its dileucine motif and, contradictorily, activates the infected cells through TCR signaling [52]. Conversely, Vpu protein downregulates TIM-3 surface expression on infected CD4^+^ T cells via its transmembrane domain and alters its subcellular localization [53]. These effects might be due to the early expression of Nef, which favors viral replication, while Vpu is expressed late to facilitate viral release.

In progressive HIV infection, high expression of TIM-3 on HIV-1-specific CD8^+^ T cells is correlated with the frequency of a dysfunctional T cell population [15]. Treg cells utilize the TIM-3/galectin-9 pathway to suppress proliferation of HIV-specific CD8^+^ T cells; the protective allele HLA-B*27- and HLA-B*5-restricted CD8^+^ T cells present a low level of TIM-3 upregulation that can evade the Treg cell-mediated suppression [54]. This explains why HIV elite controllers possessing these HLA alleles are able to maintain functional HIV-specific CD8^+^ T cells, which are responsible for delayed HIV progression [54]. Nevertheless, blocking the TIM-3 signaling pathway could restore HIV-1-specific CD8^+^ T cell proliferation and cytotoxic capabilities [15,55].

### 2.4. LAG-3

LAG-3, also known as CD223, is an immunomodulatory molecule expressed on the surface of active T cells that interacts with the TCR/CD3 complex and transduces an inhibitory signal to suppress T cell responses [56]. Significant upregulation of LAG-3 has been observed in PLWH, particularly in the activated, effector memory, and fully differentiated memory subsets of CD4^+^ and CD8^+^ T cells, which can be reduced effectively by HAART [13,18]. It is also associated with the plasma HIV viral load and disease progression [57]. Overexpression of LAG-3 in vitro in Jurkat cells causes a severe reduction in pro-inflammatory IL-2 and IFN-γ production, and ex vivo administration of LAG-3–Fc fusion protein, which disrupts the interaction of LAG-3 with its MHC II ligands, augments IFN-γ production and proliferation of HIV-specific CD8^+^ and CD4^+^ T cells [13].

LAG-3 could act synergistically with PD-1 to regulate T cell-mediated immunity [58]. Among CD8^+^ T cell populations, the PD-1^High (Hi)^ LAG-3^+^ subset has the lowest potential in producing cytokines and performing cytolytic activity, indicating severely impaired T cell function [59]. During chronic HBV and LCMV infection, the functionality of exhausted CD4^+^ and CD8^+^ T cells with high PD-1 and LAG-3 could be partially restored after blocking both inhibitory receptors [60,61,62]. Limited evidence of LAG-3 blockade has been shown in the case of HIV infection. 

### 2.5. TIGIT

TIGIT is a co-inhibitory molecule that is expressed on different T cell subpopulations, including active, memory, exhausted, follicular helper, natural killer (NK), and Treg cells [63,64,65]. TIGIT attenuates the TCR signal and induces the release of anti-inflammatory IL-10 while inhibiting the production of pro-inflammatory IL-12; TIGIT-knockout mice generate high levels of pro-inflammatory cytokines and a reduced level of IL-10 compared to their wild-type counterparts [66,67]. 

Indeed, increased expression of TIGIT has been detected on HIV-specific CD8^+^ T cells with poor functionality in PLWH, suggesting this subset represents an exhausted phenotype [68]. In accordance with the finding, a recent study demonstrates that the TIGIT^+^ NK cells derived from PLWH lose the capability to produce TNF-α, IFN-γ, and CD107a, which are positively associated with viral load [69]. Co-expression of PD-1, TIGIT, and LAG-3 can be determined as markers of dysfunctional HIV-specific T cells during viral persistence [16,18]. In CD8^+^ T cells derived from cancer patients, TIGIT is upregulated along with PD-1, and combination therapy invariably results in a significant outcome of tumor growth inhibition [70,71]. A recent study demonstrates that the combination blockade of LAG-3, CTLA-4, or TIGIT increases the frequency of cells expressing CD107a and IL-2, which are associated with cytotoxicity and survival of HIV-specific CD4^+^ and CD8^+^ T cells in HAART-treated PLWH [72].

## 3. Metabolic Plasticity in T Cells

Cellular metabolism plays a fundamental role in balancing the nutrient consumption and energy production of a cell to suit the dynamic changes of an intracellular or extracellular environment. Glucose serves as an important energy source for T cells, and a lack of glucose compromises T cell functions [73,74]. The main routes of glucose metabolism comprise glycolysis, the tricarboxylic acid (TCA) cycle, and oxidative phosphorylation (OXPHOS) [75,76]. T cells adapt to different mechanisms to oxidize glucose to support the biosynthetic demands, which are integrally linked to T cell proliferation, differentiation, and cellular functions.

### 3.1. Activated T Cells Utilize Aerobic Glycolysis

**Figure 1:**
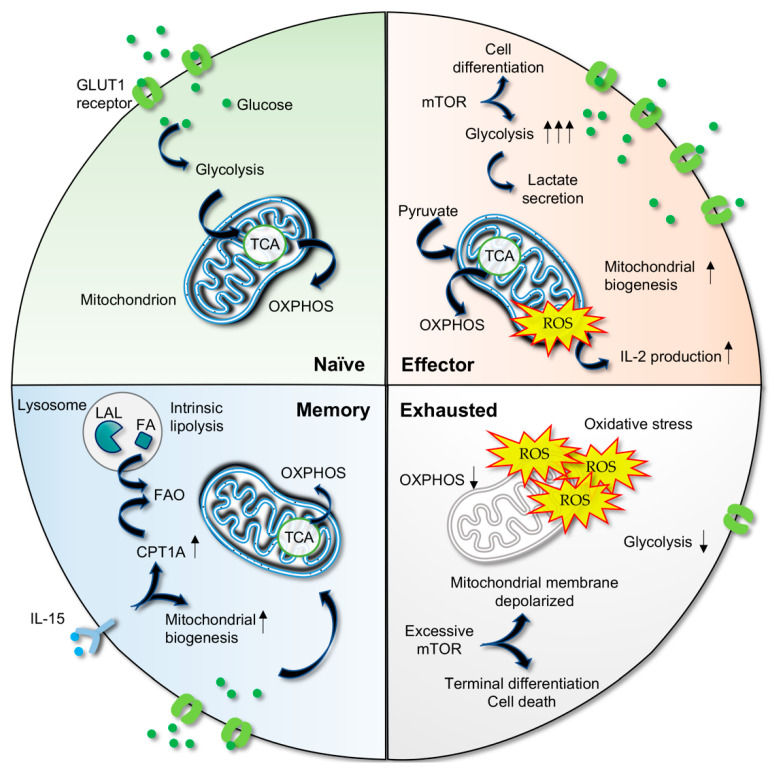
Cellular metabolism in different states of cell-mediated responses. Naive T cells utilize glucose and generate adenosine triphosphate (ATP) using oxidative phosphorylation (OXPHOS). During the transition from naïve to effector cells, glucose transporter 1 (GLUT1) receptors are increased, followed by elevated glucose uptake. The mammalian target of rapamycin (mTOR) signaling pathway assists in the increased glycolytic and lactate pathway activities to sustain cell proliferation, differentiation, and effector functions. Pyruvate, in the absence of glucose, enters the tricarboxylic acid (TCA) cycle and mediates OXPHOS. These result in increased mitochondrial biogenesis and reactive oxygen species (ROS) levels, which are important for interleukin-2 (IL-2) production. In an exhausted T cell, glucose metabolism is decreased, as shown by the reduction of the GLUT1 receptor, glycolysis, OXPHOS, and ATP production. Excessive mTOR signaling in exhausted T cells results in terminal differentiation of T cells, depolarized mitochondrial membrane, and cell death. Accumulation of a high ROS level leads to oxidative stress and negatively impacts OXPHOS efficiency as well as other metabolisms that depend on ATP. In memory T cells, metabolism is shifted back to OXPHOS and fatty acid oxidation (FAO). IL-15 plays a role in enhancing the mitochondrial biogenesis and the carnitine palmitoyl transferase IA (CPT1A) enzyme to facilitate FAO. The supply of fatty acids (FA) for FAO is acquired from intrinsic lipolysis by lysosomal acid lipase (LAL).

Under normal circumstances, naïve T cells utilize OXPHOS, which is the most efficient energy production route (Figure 1). However, activation of T cells induces a switch from OXPHOS towards aerobic glycolysis in a phenomenon known as the “Warburg effect” to support rapid clonal expansion and effector function that require growth molecules such as amino acids, fatty acids, and nucleotides [77,78]. Studies demonstrate elevated glucose uptake, glycolysis, and lactate secretion [79], accompanied by high levels of expression of glycolysis-associated proteins, including glucose transporter 1 (GLUT1) and hexokinase 2 (HK2), a key enzyme engaged in glycolysis catalyzing the conversion of glucose to glucose-6-phosphate, in activated T cells [80]. The importance of glycolysis in the metabolic remodeling of T cells is further highlighted by diminished T cell viability and immunological function in the absence of glycolysis [81]. 

Both aerobic glycolysis and OXPHOS can be utilized by proliferating cells in an activation state [82]. Upon TCR stimulation, activated T cells upregulate complex IV (cytochrome c oxidase) in OXPHOS [83] and promote mitochondrial biogenesis to sustain cellular energy demands [84]. Sena et al. have shown that pyruvate, in the absence of glucose, increases CD69 and CD25 activation markers on CD4^+^ T cells after CD3/CD28 stimulation [85] and enters the mitochondria for the TCA cycle instead of being converted to lactate [85]. Moreover, reduced mitochondria-generated reactive oxygen species (ROS) negatively impacts IL-2 production, suggesting that ROS is one of the fundamental components of T cell activation following CD3-dependent calcium influx [85]. 

Current findings also indicate that OXPHOS is required to complement glycolysis because it is capable of fulfilling cytokine production demand, sustaining proliferation and antiviral function in CD8^+^ T cells [86,87]. These findings collectively denote that mitochondrial OXPHOS is still engaged in effector activity, although glycolysis is regarded as the predominant pathway. Indeed, HIV-1 preferentially infects CD4^+^ T cells with high OXPHOS and glycolysis, as indicated by positive correlations between the metabolic activities of activated cells and HIV infection levels [88]. The activated, proliferating primary CD4^+^ T cells are highly susceptible to HIV-1 infection ex vivo for productive virus replication [89].

### 3.2. Metabolic Shift in Memory T Cells

Following viral clearance, most of the effector cells die, whereas a small number of cells from the effector population will be converted to long-lived memory T cells that possess the ability to generate rapid effector functions in response to reactivation. Along with the cellular transition from effectors with high energy demands to memory CD8^+^ T cells with a lower demand, cell metabolism is shifted back to mitochondrial OXPHOS [90,91]. 

In addition, mitochondrial fatty acid oxidation (FAO) also acts as a main energy source for memory T cells [75,90,92,93]. Memory T cells use the intrinsic lysosomal hydrolase lysosomal acid lipase (LAL) to mobilize fatty acids for the FAO process and memory cell development [90]. IL-15, a critical cytokine for memory CD8^+^ T cells, is found to regulate mitochondrial spare respiratory capacity that allows extra capacity of cells to meet the need of increased stress and hence enhances cell survival [91]. By promoting mitochondrial biogenesis and the expression of carnitine palmitoyl transferase 1A (CPT1A), which regulates FAO, IL-15 controls the bioenergetic stability of memory T cells after infection [91].

Notably, a higher frequency of central memory CD8^+^ T cells has been reported in untreated PLWH with low viral load, implying memory cells mediate effective viral control [94]. Central memory CD4^+^ and CD8^+^ T cells in PLWH express high levels of GLUT1 and mitochondrial mass [95]. Consistently, memory CD4^+^ T cells in vitro exhibit increased OXPHOS relative to aerobic glycolysis, which acts as a major determinant of HIV infection [88,96].

### 3.3. Metabolic Deregulation Results in T Cell Exhaustion

Previous interrogations of exhausted T cells isolated from chronic LCMV murine models or chronic HBV patients have revealed that the glucose metabolism is shunted away from the mitochondria, resulting in less oxidative ATP production [97,98]. Functionality of CD8^+^ T cells strongly depends on the metabolite levels of the cells, and CD8^+^ T cells display a distinct metabolomic profile prior to and upon loss of cellular functionality [99].

Metabolic dysregulation activities occur at the early stage of T cell exhaustion; these include reduced glucose uptake, glycolysis, mitochondrial respiration, and a depolarized mitochondrial membrane [97]. Chronically stimulated T cells demonstrate increased OXPHOS utilization at an early stage but the absence of this activity at a later phase, as evidenced by a significant reduction in or little consumption of mitochondrial oxygen [100]. As OXPHOS activity regulates the ROS level in mitochondria, defective mitochondrial metabolism influences the redox environment. ROS accumulation directly inflicts damage to DNA and proteins, perturbs ATP production through the inactivation of complexes on the inner membrane of the mitochondria, and reduces OXPHOS efficiency [101]. Mitochondrial dysfunction not only limits glucose oxidation; it also alters other cellular pathways that are highly dependent on ATP supply [102], adversely affects the capacity to oxidize FAO and nucleotide biosynthesis, and promotes oxidative stress that exacerbates the exhaustion [103].

In addition, the mammalian target of rapamycin (mTOR) signaling pathway persists in the effector and early exhausted T cells, and its inhibition recovers the mitochondrial fitness of early exhausted T cells, signifying its involvement in metabolic remodeling in the T cell exhaustion process [97]. It is well established that the mTORC1 pathway favors T cell differentiation into effector cells and enhances aerobic glycolysis. However, excessive mTORC1 activation results in terminally differentiated effector cells [104], mitochondrial membrane depolarization, elevated ROS levels, and cell death [105].

In chronic viral infections such as HCV, CD8^+^ T cell exhaustion is characterized by a general suppression of a broad range of genes associated with metabolic functions; these include genes encoding for mitochondrial and OXPHOS components [106]. Impaired glucose and mitochondrial metabolism, as indicated by mitochondrial membrane depolarization and a markedly high level of ROS, are detected in exhausted HCV-specific T cells. It is interesting to note that the distinct metabolic nature of the exhaustion of CD8^+^ T cells in HIV infection is different from other viral diseases. In the HIV context, glycolysis is the main energy source for CD8^+^ T cells in early, untreated PLWH and viral controllers, which is crucial for T cell killing activity. Exhausted CD8^+^ T cells show a reduction in glycolysis but increased OXPHOS capacity and a dysregulated mTOR pathway that could not be manipulated to reverse exhaustion in T cells [107].

It can be concluded that the impairment of mitochondrial OXPHOS negatively impacts T cell expansion and effector function as well as enriches the terminal differentiation phenotype, followed by oxidative stress that exerts damaging effects on the surrounding cellular components. In line with this, enhancements of OXPHOS and T cell effector function have been documented after treating exhausted T cells with antioxidants [98,106]. Collectively, current findings corroborate the perception of metabolic alterations as a driver of T cell exhaustion.

## 4. Altered Glucose Metabolism in HIV Infection

### 4.1. Glucose Metabolism Enhances Cell Permissibility to HIV Entry

**Figure 2:**
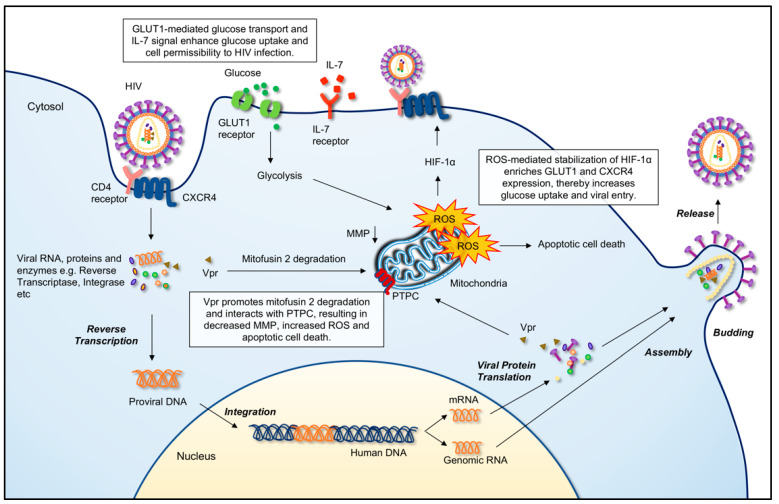
HIV infection causes metabolic alteration in the infected CD4^+^ T cell. During HIV infection, upregulation of the glucose transporter 1 (GLUT1) receptor and interleukin-7 (IL-7) signaling in T cells enhances glucose uptake and the glycolytic process, which promote viral entry through the CD4 receptor and C-X-C chemokine receptor type 4 (CXCR4). High glucose levels increase the generation of reactive oxygen species (ROS), which leads to the stabilization of the hypoxia-inducible factor 1-alpha (HIF-1α) molecule and increased expression of both GLUT1 and CXCR4, which are crucial elements in HIV infection. HIV-encoded Vpr protein promotes ubiquitin ligase degradation of the mitochondrial fusion protein mitofusin 2, causing disruption of the mitochondrial outer membrane and mitochondrial membrane potential (MMP) that leads to an increased level of ROS. Vpr also migrates to mitochondria, interacts with permeability transition pore complex (PTPC) molecules such as adenine nucleotide translocator (ANT) to form large conductance channels that trigger mitochondrial membrane permeabilization, thus resulting in a reduction of MMP and apoptotic cell death.

HIV infection results in substantial changes in the cellular metabolism of CD4^+^ T cells (Figure 2). Infection of primary CD4^+^ T cells with HIV-1 coincides with an increase in glycolysis, and upregulation of glucose uptake molecules, i.e., glucose transporters (GLUT1, GLUT3, GLUT4, and GLUT6) and hexokinase enzymes (HK1 and HK2) [108]. Studies have shown that GLUT1-mediated glucose transport is crucial in HIV infection, whereby a high glucose level significantly enhances HIV entry [109,110]. Increased GLUT1 expression in T cells following IL-7 stimulation results in augmented glucose uptake and increased permissibility to HIV-1 infection, whereas siRNA-mediated GLUT1 downregulation or signal inhibition abrogates the infection [110]. 

In HIV infection, ROS-mediated stabilization of hypoxia-inducible factor 1α (HIF-1α) results in enriched expression of GLUT1 and C-X-C chemokine receptor type 4 (CXCR4) and in CD4^+^ T cells, which thereby increases glucose uptake and viral entry [108,109,110]. On the contrary, silencing HIF-1α attenuates the expression of CXCR4 and decreases HIV entry into CD4^+^ T cells [109].

### 4.2. HIV Interferes T Cell Metabolism

Upon entry into the CD4^+^ T cell, HIV-1 replication further causes an increase in glycolytic flux whereby virion production can take place efficiently and eventually enhances virus-induced cell death in the infected cell [111]. Several key metabolites involved in glycolysis, such as hexose-P, fructose 1,6-bisphosphate (FBP), glyceraldehyde-3P (G3P), and 3-phosphoglycerate (3PG), are evidently elevated in infected CD4^+^ T cells [112]. Moreover, suboptimal inhibition of glycolysis eliminates HIV-infected cells and impairs viral amplification in CD4^+^ T cells isolated from HAART-treated PLWH [88].

Stimulation of HIV-encoded Vpr in human CD4^+^ T cells causes a dramatic reduction of mitochondrial membrane potential (MMP) and apoptotic cell death [113]. HIV Vpr protein interferes with mitochondrial outer membrane and MMP by promoting ubiquitin ligase degradation of mitochondrial fusion protein mitofusin 2, which leads to increased mitochondrial ROS levels [114]. Vpr also interacts with the adenine nucleotide translocator (ANT), a mitochondrial membrane protein that cooperate within the permeability of the transition pore complex (PTPC), to form large conductance channels that trigger mitochondrial membrane permeabilization and cell death [115].

### 4.3. Long-Term HAART Positively Influences T Cell Metabolism Fitness

In addition to HIV, HAART treatment causes T cell metabolic alteration in PLWH. The older generation of HAART treatment, in particular nucleoside reverse transcriptase inhibitors, triggers severe mitochondrial toxicity through inhibition of DNA polymerase gamma which is followed by the depletion of mitochondrial DNA [116]. While the newer treatment with different classes of HAART drugs is able to improve metabolic fitness in CD8^+^ T cells, it is unable to restore mitochondrial impairment [117]. Robustly functional mitochondria are coupled with a healthy bioenergetic phenotype and have a direct role in cellular exhaustion. Enforcement of metabolic fitness at an early phase of infection is important to counteract full scale cell exhaustion and is therefore crucial for repressing disease progression. Apart from that, early initiation of HAART allows rapid and close-to-complete normalization of T cell subsets and preserves T cell functions [118], as well as preventing metabolic alterations that drive T cell exhaustion. 

HIV-1-specific CD8^+^ T cells from PLWH receiving long term HAART for more than 10 years demonstrate improved polyfunctionality and capacity to eliminate target cells in vitro, compared to the CD8^+^ T cells from PLWH on short term HAART [119]. This improvement is attributable to differences in the frequencies of central memory CD8^+^ T cells, reduced mitochondrial respiration, and glycolytic induction upon TCR activation. Further, CD4^+^ T cells derived from individuals receiving integrase strand transfer inhibitors such as dolutegravir or elvitegravir, exhibit a significant reduction in ex vivo proliferative capacity, supporting the notion that cellular metabolism and functionality are influenced by external factors, including drugs [117].

### 4.4. T Cell Metabolism in Elite Controllers

Elite controllers represent <1% of PLWH who can naturally suppress the viral infection in the absence of HAART therapy. Investigation of the plasma metabolites from the elite controller cohort without treatment intervention demonstrates that the transient controller subgroup, which eventually lost virological control within 12 months but not the persistent controller subgroup, is profiled by increased aerobic glycolysis, dysfunctional mitochondria, and a high level of oxidative stress [99]. Persistent immune activation and cytokine production are also detected in the transient controllers and strongly associated with the metabolite level [99], while the opposite trends are observed in the persistent controllers [120]. This suggests an intact mitochondrial function contributes to a greater control of the virus in PLWH. 

Studies have shown that the mTOR pathway is upregulated in elite controllers and dysregulated between males and females in the elite controller cohort, indicating an imperative role in controlling latent HIV infection [121,122,123]. mTOR complex subunits knocked down by CRIPSR interference or pharmacological inhibition, as well as inhibition of Enolase 1 in CD4^+^ T cells, prevent viral reactivation and suppress latency reversal by repressing Tat-dependent and Tat-independent transcription of HIV [122].

The mTOR pathway regulates HIV-1 latency through its downstream pathways, such as HIF signaling. HIF signaling is a major regulator of cellular metabolism, which assists cellular adaptation to oxygen availability, growth factors, cytokines, and infections via mTOR. Hence, activation of HIF signaling results in the regulation of numerous downstream target genes mediating cell survival, proliferation, immune function, as well as metabolic reprogramming [124]. Interestingly, a recent study utilizing proteo-transcriptomic data integration suggests enriched HIF signaling, a high ratio of HIF-1α and HIF-1β nuclear localization, and glycolysis are unique features of the male elite controllers [121].

By comparing the immunometabolic profiles of HIV-specific CD8^+^ T cells between elite controllers and non-controllers (HAART-treated individuals), a greater metabolic plasticity of CD8^+^ T cells is observed in the HIV controllers [125]. Furthermore, while elite controllers possess HIV-specific CD8^+^ T cells that appear to be mitochondrial-dependent to fuel T cell responses in addition to glycolysis, HIV-specific CD8^+^ T cells from non-controllers show larger mitochondrial mass, which has been linked to dysfunctional mitochondria [125].

## 5. Achieving HIV Remission through Immune Checkpoint Inhibitors and Glucose Metabolic Enhancement

Immune checkpoint inhibitors are often defined as effective immunotherapies for various cancers and chronic infections. Emerging evidence supports the notion that metabolic reprogramming has a direct impact on T cell differentiation and exhaustion [78,85,126]. In HIV infection, chronically stimulated T cells display increased PD-1 expression accompanied by a significant reduction of cellular respiration [117]. It is interesting to note that metabolic activities vary between PD-1^Intermediate (Int)^ and PD-1^Hi^ exhausted T cell subsets; the PD-1^Int^ subset displays a high rate of glucose uptake and OXPHOS and low mTOR activity and mitochondrial mass, whereas the PD-1^Hi^ subset shows the opposite [97], which highlights a close relationship between cellular metabolism and immunoregulation. The effects of PD-1 on the metabolic functions and bioenergetics of activated CD4^+^ T cells have also been examined. Patsoukis et al. have revealed ex vivo PD-1^+^ T cells could not uptake and utilize glucose, as shown by the inhibition of GLUT1 expression, glucose transport, and *HK2* mRNA levels in T cells upon receiving PD-1 signals [127].

Since upregulation of immune checkpoints in T cells derived from PLWH is positively correlated with metabolic dysregulation, immune checkpoint inhibitors such as anti-PD-1 have been suggested to rescue CD8^+^ T cells from exhaustion status. Nevertheless, there is still limited clinical data regarding the immune checkpoint antibodies and their impacts on T cell metabolic functions in an HIV context. Anti-PD-1 antibody treatment partially alleviates the progression of cellular exhaustion by enhancing OXPHOS, glycolysis, the IL-2 signaling pathway, and T cell effector function [97,98,106,128]. In addition, PD-1 suppresses peroxisome proliferator-activated receptor gamma coactivator 1-alpha (PGC-1α), a key metabolic regulator and transcriptional co-activator [97,129], which functions to maintain mitochondrial fitness in tumor-infiltrating lymphocytes [130], whereas blockade of the PD-1 signal enables PGC-1α activity to enhance glucose uptake and partially reverse the metabolic defects. These findings emphasize the efficiency of PD-1 inhibition on the restoration of metabolic fitness and, subsequently, T cell activation status.

Additionally, co-expression of PD-1 and CTLA-4 acts cooperatively to reduce cellular glucose uptake and causes glucose metabolic derangement, which is coherent with the progressive loss of T cell effector functions during viral antigen persistence [100]. Co-inhibitory receptor treatment targeting CTLA-4 and PD-1 induces a synergistic pattern of metabolic and effector T cell-specific functions, including enhancement of effector molecules such as IFN-γ, Granzyme A, Granzyme B, and Fas ligand production [128]. Treatment with a dual checkpoint blockade serves a more potent strategy by targeting different pathways that enhance antiviral responses and T cell metabolism.

Considering the effect of HAART on immune checkpoint expression, studies have combined multiple therapies to investigate T cell function and the reversal of HIV latency. PD-1 inhibition along with HAART reprofiles the CD8^+^ T cells with augmented antiviral responses, including perforin and granzyme B production, as well as viral reservoir depletion in the chronically SIV-infected rhesus macaque [131]. Treatment of CD8^+^ T cells from PLWH with pembrolizumab (anti-PD-1) and the latency reversing agent bryostatin reduces the latent reservoir in vivo without enhancing T cell activation [132]. Therefore, combined treatment, including immune checkpoint inhibitors, remains a possible solution to achieve HIV remission.

The study has also demonstrated a higher frequency of TIM3^+^ PD1^-^ CD8^+^ T cells in long term HAART recipients as compared to higher PD1^+^ TIGIT^+^ cells in short-term HAART recipients. As such, high efficiency in restoring the cytotoxic capacities of CD8^+^ T cells has been reported upon combined treatment of pro-glycolytic drugs such as metformin with anti-TIGIT and anti-PD-1 antibodies in vitro [119].

Intriguingly, metformin (also an anti-diabetic drug) reduces the frequency of PD1^+^ TIM3^+^ TIGIT^+^-expressing CD4^+^ T cells in HAART-treated PLWH [133]. A recent study demonstrates that CD4^+^ T cells from women PLWH with diabetes mellitus receiving metformin show lower glucose metabolic activity as compared to those without medication [134]. Thus, it is suggested that metformin could be an adjunct therapy to reduce metabolic activation, partially correct glucose metabolism in CD4^+^ T cells, and well as reverse exhaustion [135].

## 6. Conclusions

T cell function and exhaustion are intimately linked to metabolic changes within cells. In chronic HIV infection, increased immune checkpoint molecules and an altered bioenergetic profile result in cellular exhaustion. The use of immune checkpoint inhibitors coupled with metabolism-enhancing drugs to restore T cell exhaustion serve as promising complementary therapeutic options for HIV. Therefore, further research is warranted to uncover the complex mechanism underlying metabolic changes in mitochondrial pathways that are intricately concomitant with immune cell exhaustion in chronic HIV infection.
